# Management of hospital-acquired infections among patients hospitalized at Zewditu memorial hospital, Addis Ababa, Ethiopia: A prospective cross-sectional study

**DOI:** 10.1371/journal.pone.0231949

**Published:** 2020-04-24

**Authors:** Segen Gebremeskel Tassew, Minyahil Alebachew Woldu, Wondwossen Amogne Degu, Workineh Shibeshi

**Affiliations:** 1 Department of Clinical Pharmacy, School of Pharmacy, College of Health Sciences, Mekelle University, Mekelle, Ethiopia; 2 Department of Pharmacology and Clinical Pharmacy, School of Pharmacy, College of Health Sciences, Addis Ababa University, Addis Ababa, Ethiopia; 3 Department of Internal Medicine, School of Medicine, College of Health Sciences, Addis Ababa University, Addis Ababa, Ethiopia; University of Malta Faculty of Health Sciences, MALTA

## Abstract

**Background:**

Hospital-Acquired Infections (HAIs) are acquired when the patient is hospitalized for more than 48 hours. In Ethiopia data are scarce in management appropriateness of HAIs. Hence, this study was aimed to assess the prevalence and management of HAIs among patients admitted at Zewditu Memorial Hospital.

**Method:**

A facility based prospective cross sectional study was conducted from March 1, 2017 to August 30, 2017. The sample was proportionally allocated among (medical, pediatrics, gynecology and obstetrics and surgical) wards, based on patient flow. Data were collected using data abstraction format and supplemented by key informant interview. Interview was made on eight physicians and four microbiologists who have been working in the wards during study period. Management appropriateness was assessed using Infectious Disease Society of America guideline and experts opinion (Infectious disease specialist). A multivariate logistic regression was used to identify factors associated with HAIs.

**Result:**

The prevalence of HAIs was 19.8%. Surgical Site Infection (SSI) and pneumonia accounted for 20 (24.7%) of the infections. Culture and sensitivity was done for 24 (29.6%) patients. Of the 81 patients who developed HAIs, 54 (66.67%) of them were treated inappropriately. Physicians’ response for this variation was information gap, forgetfulness, affordability and availability issue of first line medications. Younger age (AOR (Adjusted odds ratio) = 8.53, 95% CI: 2.67–27.30); male gender (AOR = 2.06, 95% CI: 1.01–4.22); longer hospital stay (AOR = 0.17, 95% CI: 0.06–0.51); and previous hospital admission (AOR = 3.22, 95% CI: 1.76–5.89); were independent predictors of HAIs.

**Conclusion:**

Prevalence of HAIs and inappropriate management were substantially high in this study. Pneumonia and SSI were the common types of HAIs. Locally conformable guidelines could help to correct such problems.

## Introduction

Hospital-acquired Infections (HAIs) or nosocomial infections are defined as infections which are not present or not incubating when the patient is hospitalized and are acquired after 48 hours of hospital stay [1]. In developing countries large proportion of people are dying on daily preventable and curable diseases due to inadequate health care services in which postoperative HAIs constitute a large proportion of this burden in which increasing its risk by nine times more than the developed countries [2,3]. Studies in Ethiopia focusing only on surgical and gynecologic/obstetrics wards showed prevalence of HAIs as high as 27.6%. The risk of HAIs in relation to surgery is high, since about 77% of death of patients with HAIs was reported to be related with postoperative infections [4].

HAIs prolong hospital stays, create long-term disability, increased patient morbidity and mortality, increase resistance to antimicrobials and represent a massive additional financial burden for health systems [5–7]. Such infections annually account for 37, 000 attributable deaths in Europe and they account for 99,000 deaths in the USA, reflecting 16 million extra days of hospital stay. Patients who developed HAIs which were admitted at ICU had higher mortality rate as compared to patients who did not acquired HAIs [8,9].

In Pakistan, it has been reported that due to resistance to first line antibiotics, 70% of HAIs could not be successfully treated by using WHO’s recommended regimen [10]. A retrospective cohort A study done at a single academic medical center among patients hospitalized in Chicago, Illinois 38.2% of patients received inappropriate empiric therapy [11]. A retrospective study carried among 286 patients, 151 (52.8%) patients received inappropriate therapy [12].

In developing countries prevalence data are often not well established [13]. The traditional approach to empiric treatment is to start with inexpensive narrow-spectrum antibiotics and change it to broad-spectrum antibiotics if a multi-resistant pathogen is identified or the patient deteriorates. However, inadequate empiric therapy has been shown to cost both lives and money [14].

In Ethiopia, regardless of the growing burden of various types of HAIs; it continues to receive relatively low public health priority. HAIs have been remaining a public health issue because of its devastating effect and double burden for both patients and their families in general, however, there are no researches done focusing on the management appropriateness of HAIs. Therefore, there was a clear need to conduct such research to explore the prevalence and management appropriateness of the problem. This research work was intended to contribute to the appropriate management of HAIs and will benefit both patients and health care professionals. Successful treatment is achieved by the initial use of the correct antimicrobial agent at the most appropriate dose to optimize the likelihood of clinical and bacteriological success and minimize drug-related toxicities. Therefore, this research will assist physicians in the selection of an appropriate choice of antibiotics to avoid the emergence of multidrug-resistant organisms in hospitals.

## Material and methods

### Study design and setting

A hospital based prospective cross sectional study design was conducted from March 1, 2017 to August 30, 2017. Both quantitative and qualitative methods were used. Patients admitted and stayed for more than 48 hours were used as research participants. Key informant interview with the help of semi-structured open ended questionnaire focusing on the experience and practice of practitioners (Eight physicians) regarding management and guidelines used to treat HAIs were used. Microbiologists were also interviewed regarding the laboratory concerns (Four personnel). All senior physicians and microbiologists who were working on the wards during the study period were used in the key informant interview. The qualitative data were done after the results to enrich the results obtained from patient’s chart. This study was conducted at Zewditu Memorial Hospital, which is a teaching and general referral hospital affiliated to College of Health Sciences, Addis Ababa University.

### Ethical statement

Ethical clearance was obtained from Ethics Review Board of the School of Pharmacy; College of Health Sciences, Addis Ababa University. Permission was also obtained from hospital administration of Zewditu Memorial Hospital to conduct the study and to access the medical record. Written informed consent was prepared to insure patients understanding and willingness to participate in the study. Consent was obtained from care givers for those severely ill patients and children. Finally verbal consent was given for physicians and microbiologists and their response was recorded using audio recorder.

### Target and study population

The target population was all patients admitted in medical, pediatrics, gyn/obs and surgical wards of ZMH (Zewditu memorial hospital) during the study period. The study population was a target population who fulfilled the inclusion criteria of this study.

### Inclusion and exclusion criteria

#### Inclusion criteria

Patients admitted in selected wards of Zewditu memorial hospital and who have stayed in the hospital for at least 48 hours, Patients readmitted within 30 days of discharge after surgery and for those who did not undergo surgery readmission within 3 days of discharge.

#### Exclusion criteria

Admitted patient with incomplete medical records were excluded for the study.

### Sample size and sampling procedures

The sample size required was calculated using a single proportion sample size estimating formula [15].

n=(Zα2)2P(1−P)d2

(Where n = required initial sample size, *Z α*/2 = critical value for normal distribution at 95% confidence interval which equals 1.96 (*Z* value at alpha = 0.05), *P* = proportion of population having HAI (p = 0.5), and *d* = marginal error (5% = 0.05).

Calculation provided ‘n’ value of 384. Considering 7% contingency (for non-response rate), the final sample size of the study became 410. Therefore, the sample size was allocated to the wards using proportion to size allocation (Medical 54, pediatrics 88, gyn/obs 156 and surgical ward 112) and these patients were followed prospectively.

### Dependent and independent variables

#### Dependent variables

Hospital-acquired infections (HAIs were defined using CDC guideline.) such as Hospital Acquired Pneumonia (HAP), Surgical site infections (SSIs), Urinary tract Infections (UTIs), meningitis, sepsis, ventriculitis, Gastrointestinal system infections and Skin and soft tissue infections and management appropriateness (Appropriateness of management was checked by IDSA guidelines and experts opinion), such as right choice of medication and dosage form, route of administration, dose, frequency, duration and drug interaction were the outcome (dependent) variables.

#### Independent variables

Age, Gender, Ward type, Patients on immunosuppressive agents, Length of hospital stay, Previous hospital admission, Exposures to invasive devices (urinary catheter, peripheral intravascular catheter and mechanical ventilator), Presence of chronic disease, Type and Duration of procedure, History of previous surgery, Use of proton Pump inhibitors, Use of antacid, use of Steroids, Culture and Sensitivity test, Indication for use, Dose, Frequency of administration and Duration of treatment were the independent variables studied.

### Data collection tools and techniques

The data abstraction format was prepared in English which is adopted from previous studies and some modifications were done by infectious disease specialist and pharmacists. Data was collected by four nurses from the respective wards using structured data abstraction format from patient medical charts. The data collectors were given training course of a day by the principal investigator. The data collectors reviewed each patient’s medical records after two days of admission and daily thereafter during the entire inpatient stay for any new medical records. The data abstraction was designed to record socio demographic and clinical characteristics of study participants, types of HAIs, culture and sensitivity and type of medications given.

Appropriateness of the management was assessed using expert’s opinion and infectious disease society of America guidelines focusing on the medication, dosage form, dose, frequency, rout of administration, duration of treatment for HAIs. Micromedex^®^ was used to check drug interactions [16–18].

Interview of professionals using key informant interview using semi-structured open ended questionnaire was used to assess the experience and practice of the practitioners regarding the diagnosis and management of HAIs was done by principal investigator.

The interview assessed back ground information including education level and current responsibilities and question regarding patient assessment, medication choice and specific guidelines they follow if there is any. Finally, issues on barriers to optimal treatment of HAIs from the physicians’ perspective were assessed. Microbiologists were interviewed focusing on the quality of the laboratory and the time required for the results.

Data was collected by data collectors, to ensure the validity and accuracy of the data pretest was done on 23 patients prior to the actual data collection to assess the data collection tool. The pre tested patients were not included in the analysis. Based on the finding amendments and arrangements was made on the data collection tool. The collected data was supervised by principal investigator every day and corrected immediately (if any). Double data entry was conducted by principal investigator to minimize errors.

### Definitions

#### Hospital Acquired Infection (HAI)

Is defined as HAI when it originated in the hospital environment after 48 hours of admission to hospital and the types of infections were classified according to CDC guideline [1].

#### Appropriate use of antibiotic

The indication for use, dose, and frequency of administration, duration of treatment, no or mild drug interaction and Culture and sensitivity (C&S) investigation were according to guidelines recommendations for treating HAIs.

#### Inappropriate use of antibiotic

The indication for use, dose, frequency of administration, duration of treatment and culture and sensitivity investigation were not according to guidelines recommendations for treating HAIs.

### Data analysis and interpretation

The collected data were coded, entered into EpiInfo^™^ Manager Version 4.0.2., and analysis was done using the SPSS version 21. Descriptive analysis was made to describe the study variables and presented using percentages and median. Logistic regression was used to assess factors associated with the development of HAIs. Adjusted odds ratio with its 95% CI was used to assess the prevalence of association between each of the independent variable and outcome of interest. Variables with p≤0.20 in bivariate analysis were taken for multivariate analysis. Significance was declared at p-value ≤ 0.05.

## Results

### Socio demographic characteristics

A total of 410 patients were included giving a response rate of 97%. The median age of the patients was 28 years and 199 (48.5%) of them were aged between 15–34 years. More than half of the participants were female accounting for 277 (67.6%). Majority of the patients 156 (38.0%) were form Obstetrics and gynecology ward (Table 1).

**Table 1:** The socio-demographic characteristics of patients admitted in Zewditu memorial hospital from March 1, 2017 -August, 2017.

Characteristics	Number of patients	Percent
**Sex**		
** Male**	133	32.4
** Female**	277	67.6
**Age (years)**		
** <1**	35	8.5
** 1–14**	55	13.4
** 15–34**	199	48.5
** 35–55**	79	19.3
** >56**	42	10.2
**Ward type**		
** Medical**	54	13.2
** Surgical**	112	27.3
** Pediatrics**	88	21.5
** Obstetrics and gynecology**	156	38.0

### Clinical characteristics

Majority of 136 (33.2%) admitted patients stayed in hospital for about five to ten days. The mean (SD) length of hospital stay was 11.1(8.7). From the ninety seven (23.6%) patients who had chronic disease 27(6.6%) of them had HIV followed by HTN 26(6.3%). From the total of 264 patients who underwent surgery 67 (25.4%) of them had clean and 176 (66.7%) had clean contaminated type of surgery in which the procedure took 31 to 60 minutes moreover the average duration of surgical procedure was 42.7 minutes More than half 150 (56.6%) of the procedures were elective surgery in which majority of the patients stayed for less than seven days preoperatively. Two hundred fifty four (95.8%) of the patients had received preoperative antibiotics prophylaxis (Table 2).

**Table 2:** Clinical characteristics of patients admitted in Zewditu memorial hospital from March 1, 2017-August 30, 2017.

Characteristics	Number of patients (N)	Percentage
**Hospital stay(days)**		
** <5**	124	30.2
** 5–10**	136	33.2
** 11–15**	64	15.6
** 16–21**	38	9.3
** >21**	48	11.7
**Chronic disease**		
** Yes**	97	23.6
** No**	313	76.4
**Types of chronic disease**		
** HIV**	27	6.6
** DM**	15	3.7
** HTN**	26	6.3
** CKD**	9	2.2
** CHF**	15	3.7
** Asthma**	5	1.2
**Peripheral line inserted**		
** Yes**	382	93.2
** No**	28	6.8
**Catheterized**		
** Yes**	174	42.4
** No**	236	57.6
**NGT inserted**		
** Yes**	59	14.4
** No**	351	85.6
**Mechanical ventilation**		
** Yes**	14	3.4
** No**	396	96.6
**Use of PPI**		
** Yes**	8	2.0
** No**	402	98.0
**Use of antacid**		
** Yes**	14	3.4
** No**	396	96.6
**Lumbar puncture done**		
** Yes**	15	3.7
** No**	395	96.3
**Surgery**		
** Yes**	264	64.4
** No**	146	35.6
** Elective**	150	56.8
** Emergency**	114	43.2
**Pre-operative prophylaxis**		
** Yes**	253	95.8
** No**	11	4.2
**Wound class**		
** Clean**	67	25.4
** Clean contaminated**	176	66.7
** Contaminated**	17	6.4
** Dirty**	4	1.5
**Preoperative hospital stay (days)**		
** <7**	138	52.2
** >7**	126	47.8
**Previous surgery(n = 264)**		
** Yes**	42	15.9
** No**	222	84.1
**Duration of procedure (minutes)**		
** <30**	60	22.7
** 31–60**	124	46.9
** 61–90**	34	12.8
** 91–150**	36	13.8
** >150**	10	3.8

CKD: Chronic kidney disease, HTN: Hypertension, HIV: Human immune virus, DM: Diabetes mellitus, CHF: Congestive heart failure, NGT: Nasogastric tube, PPI: Proton pump inhibitors.

#### Proportion of specific infections among hospital-acquired infections

A total of 81 (19.8%) patients experienced HAI. In addition, 22 (27.2%) patients suffered from more than one types of HAIs. Majority of infections were pneumonia and Surgical Site Infection (SSI) in 20 (24.7%) of the cases, followed by Urinary Tract Infection (UTI) in 16 (19.8%). According to the key informant result, six of the respondents agreed that there is poor infection prevention practice in the setup. Their main reason for the poor practice was due to shortage of time and negligence. The other reason that was stated was patients were treated empirically without supportive laboratory evidences this can contribute to the overestimated prevalence. (Table 3).

**Table 3:** Proportion of specific site infections among patients admitted in Zewditu memorial hospital from March 1, 2017-August, 2017.

Characteristics^a^	Number of patients (ward)				Percentage
	Medical	Pediatrics	Surgical	Gyn/obs	
**Surgical site infections**	-	9	5	6	24.7%
**Pneumonia**	14	4	2	-	24.7%
**Urinary tract infections**	6	4	3	3	19.8%
**Sepsis**	4	4	3	5	17.5%
**Ventriculitis**	3	4	3	2	15%
**Gastrointestinal system infections**	4	6	-	-	12.7%
**Meningitis**	-	8	-	-	10%
**Skin and soft tissue infection**	4	-	-	-	5.1%
**Others**	4	3	-	-	8.8%

^a^ One patient can have more than one infection

Others: Unspecified HAIs

#### Associated factors for the development of hospital-acquired infections

Bivariate analysis indicated that patients admitted in pediatrics ward were relatively at risk of developing HAIs as compared to gynecological ward (p = **<0.001**). Patients whose duration of procedure from 30–60 minutes have relatively less risk of developing HAIs (12%) and it was statically significant (p = 0.001) as compared to duration of procedure >150 minutes. Patients who had clean contaminated surgery were at higher risk of developing HAIs as compared to patients who undergo clean surgery.

Emergency surgery puts patients more at risk (65%) of infection as compared to these patients who had elective surgery and statistically significance were observed (p = 0.004). Patients who took preoperative prophylaxis had less risk (13.8%) of developing HAIs as compared to these who didn’t took preoperative prophylaxis (P = 0.009).

Multivariable logistic regression analyses were conducted to explore the association between dependent and independent variables. Independent variables were younger age, male gender, longer hospital stay and previous admission. The chance of developing HAIs among patients less than one year was 8.5 times higher as compared to age 56 and above (AOR = 8.53,95% CI: 2.67–27.30). Compared to females males had 2.07 higher odds of developing HAIs (AOR = 2.06, 95% CI: 1.01–4.22). Patients admitted for less than five days had 96.8% less risk of developing HAIs as compared to these who stayed for greater than 21days (AOR = 0.32, 95% CI: 0.01–0.10). The odds of developing HAIs among patients who had lumbar puncture procedure were 24.98 times higher than patients who didn’t had lumbar puncture as a procedure (AOR = 24.98, 95% CI: 4.17–149.28). Patients who had history of previous admission were 3.22 times at risk of developing HAIs as compared to patients who had no any history of previous admission (AOR = 3.22, 95% CI: 1.76–5.89) (Table 4).

**Table 4:** Multivariate analysis of factors associated with hospital-acquired infections among patients admitted in Zewditu memorial hospital, March 1,2017 -August, 2017.

		HAIs		COR	pvalue	AOR (95% CI)	pvalue
Characteristics		Yes	No	(95% CI)			
**Sex**	Male	38(28.6)	95(71.4)	0.45(0.27–0.75)	**0.002**	2.06(1.01–4.22)	**0.047**
	Female	43(15.5)	234(84.5)	1		1	
**Age**	<1	19(54.3)	16(45.7)	7.12(2.39–21.20)	0.130	8.53(2.67–27.30)	**<0.001**
	1–14	15(27.3)	40(72.7)	2.25(0.78–6.41)	0.772	2.46(0.83–7.33)	0.104
	15–34	32(16.1)	167(83.9)	1.15(0.44–2.95)	0.812	1.78(0.64–4.96)	0.266
	35–55	9(11.4)	70(88.6)	0.77(0.25–2.33)	0.646	1.01(0.32–3.16)	0.978
	>56	6(14.3)	36(85.7)	1		1	
**Ward**	Medical	17(31.5)	37(68.5)	3.52(1.65–7.50)	0.001	2.05(0.77–5.46)	0.148
	Surgical	12(10.7)	100(89.3)	0.92 (0.42–1.99)	0.833	0.78(0.30–2.05)	0.626
	Pediatrics	34(38.6)	54(61.4)	4.82(2.51–9.26)	**<0.001**	0.40(0.12–1.30)	0.130
	Gyn/obs	18(11.5)	138(88.5)	1		1	
**Hospital stay(days)**	<5	5(6.2%)	119(36.2%)	0.23(0.08–0.06)	**<0.001**	0.03(0.01–0.10)	**<0.001**
	5–10	17(21.0)	119(36.2)	0.78(0.03–0.17)	**<0.001**	0.09(0.03–0.23)	**<0.001**
	11–15	18(22.2)	46(14.0)	0.21(0.09–0.48)	**<0.001**	0.27(0.11–0.67)	**0.005**
	16–21	10(12.3)	28(8.5)	0.19(0.07–0.49)	0.001	0.17(0.06–0.51)	**0.001**
	>21	31(38.3)	17(5.2%	1		1	
**Previous admission**	Yes	27(38.6)	43(61.4)	3.32(1.89–5.83)	**<0.001**	3.22(1.76–5.89)	**<0.001**
	No	54(15.9)	286(84.1)	1		1	

CKD: Chronic kidney disease, HTN: Hypertension, HIV: Human immune virus, DM: Diabetes mellitus, CHF: Congestive heart failure, PPI: Proton pump inhibitors

### Management of hospital-acquired infections

Culture and sensitivity was done for 24 (5.9%) patients who developed HAIs. About half of the specimens taken were from CSF and blood 50.0%, 33.3%, respectively. Majority of the culture and sensitivity results show no bacterial growth accounting for 17 (70.8%). From the microorganism identified, *E*.*Coli* accounted for 4 (16.7%), *S*.*aureus* for 2(8.7%) and *acinetobacter* for 1(4.2%) of the HAI (S1 File).

Key informant interview result indicated that almost all of the physicians hesitate to send culture and sensitivity test often because its time taking (Table 5), (S2 File).

**Table 5:** Responses of the interviewed physicians and microbiologists regarding the management of hospital-acquired infections among patients admitted in Zewditu memorial hospital from March 1, 2017—August, 2017.

Variables	Categories	Comments	No (%)	Percentage
Patient related factors (for inappropriate management)	Compliance	Patients prefer PO than injections	3	37.5
	Affordability	Patients can’t afford from private pharmacies	8	100
Drug related factors	Availability	Drugs like vancomycin, ceftazidem and cefepime are not available in the hospital pharmacy all the time.	8	100
	Side effects	Vancomycin fear of nephrotoxicity in both extreme ages.	2	25
Institution related factors	Guideline	No specific guideline including STG for Ethiopia doesn’t have treatment protocol for HAIs	8	100
	Clinic setup	Limited laboratory reagents and disks. More Patient flow and limited number of beds. Culture will take several days difficult to manage patients.	6	75
Physician related factors	Information gap	This inappropriateness could be due to information gap on updated recommendation.	8	100
	Antibiotics initiated before culture	For these patients admitted at night and weekends culture is sent after initiation of antibiotics because the laboratory is closed at that time	4	50
	Forgetfulness	Patients took antibiotics for longer duration because they are treated empirically most of the time patients remain on initiated antibiotics while adding other antibiotics on top of that. Forget to discontinue it.	4	50
	Inadequate sample	The sample sent are not adequate to detect particular pathogen like in case of hospital-acquired infections pneumonia if sent sputum.	2	50
Laboratory personnel	The quality of the laboratory is optimal to detect any growth.	The negative result may be due to Inappropriate sample collection Failure to request appropriate laboratory test Improper use of transporting medium. Sample collected after antibiotics use	4	100
	On average, how long does it take for culture and sensitivity result to come back (in day)?	Mostly 3 days Some cultures (eg. blood culture requires 7–14 days	4	100

#### Inappropriate management

Among the 81 patients who developed hospital-acquired infections, 54(66.7%) were inappropriately treated (not according to IDSA guidelines) while the rest 27(33.3%) were appropriately treated. Wrong choice of medications accounts the higher proportion 45(53.6%) followed by longer duration of antibiotics administration 16(19.0%). Using Micromedex as interaction checker no drug interaction was encountered in this study. Fifteen (18.5%) patients had change of medication the reason for change of their medication was due to poor response 46.7% followed by IV to Po change 20%, after culture and sensitivity result 20% and medication unavailability 13.3%.

The highest inappropriate management were from pediatrics ward 23 (42.6%) followed by medical ward 13 (24.1%); surgical ward 11 (20.4%); and gynecology and obstetrics ward 7 (13.0%). From the 264 patients who underwent surgery 67(25.4%) of patients took inappropriate prophylaxis for clean surgery and longer duration of postoperative prophylaxis in gyn/obs on patients who underwent C/S 104 (39%) were observed. Among patients who developed hospital-acquired pneumonia 17 (31.5%) of them were inappropriately treated followed by urinary tract infection 16 (29.6%). Majority of the respondents where giving prophylaxis for all patients because of the fear of infection since the procedure room is dirty with full of flies.

Most of the respondents to key informant interview indicated that they use international guidelines and standard text books to treat HAIs. They all agree that standard treatment guideline for Ethiopia lacks clear protocol for treatment of HAIs and they did not prefer to use it as a reference. Most of the physicians consider several factors like affordability, availability, patient compliance and possible side effects to choose medications to their patients.

## Discussion

The prevalence of HAI in our study was 19.8%. This was higher than the previous studies done in Ethiopia, Uganda and Tunisia (15.41%, 17%, and 17.9%) respectively [19–21]. This was also lower than the studies done in Lithuania 3.8% and Italy 9.8% [22,23]. This difference could be due to the differences in methodologies where they used point prevalence, the infection prevention strategies and difference in working environment. In another way, our finding was similar to the study done in Benin [24].

The most common type of HAI in our study was pneumonia and SSI (24.7%). This finding was lower than the studies conducted in Tunisia and Ethiopia [19,21,25], while this figure was higher than the study done in Hawassa, Jimma and Nigeria [21,26–28]. The reason for the difference could be the difference in inclusion criteria where patients with contaminated and dirty wound were included in these studies. Similarly, our finding was higher compared to studies done in Egypt 9.2%, Sudan 9%, USA 7.2%, and France 2.5% [29–32]. The higher SSI in our study could be due the difference in the operation room. The lack of guidelines and lack of antimicrobial stewardship in the study area could also contribute the higher prevalence. Furthermore, studies done in Egypt and Sudan included only elective surgery.

Pneumonia constituted the highest proportion of HAI according to studies done in India 50% [33] and Saudi Arabia 28.9% [34]. Our finding was lower than these studies and the reason could be these studies were conducted in ICU only. Our finding was in line with the study done in Iran where pneumonia account for 70% of cases [35].

The second most common infection encountered in our study was UTI in 19.8% of the participants and this finding was comparable with the study done in Morocco 35%, Tanzania 31.1%, Lituania 28.5%, and Benin 48.2% [24,36–38]. The reason could be majority of the admitted patients had urinary catheter during the study period. A systematic review on UTI supports our justification in that 79.3% of UTI can be prevented, if catheterization was not performed in hospitals [39].

The availability of waste disposal management system and strategies minimizing environmental exposure is believed to reduce HAIs in hospitalized patients and health care workers. A study done in Ethiopia revealed that waste management in health care facilities was poor [40].

Our finding showed that males were vulnerable to HAIs than females and this had been supported by other studies done in Nigeria [27,41]. The anticipated reasons were male patients underwent more contaminated type of procedures unlike females and catheter insertion was more common in association with prostate tumor and hypertrophy.

In our study pediatric ward admitted patients were relatively at risk of developing HAIs. Similar studies reported that the rates of neonatal infections were 3–20 times higher than those reported from hospital-borne babies in industrialized countries [42].

Patients age less than 14 years were 72.7% high likely to develop HAI compared to older age patients. This result was in line with the studies done in Nigeria and Rangpur [27,43]. This was in contrary with the previously done studies in Ethiopia and Russia [19,44]. Possible reasons for this discrepancy was that our study had relatively more pediatric patients aged less than 14 years and also there were more neurosurgical procedures.

Patients whose duration of procedure from 30–60 minutes had relatively less risk of developing HAIs as compared to duration of procedure >150 minutes. This study was supported by other studies in Ethiopia [26,27,45]. Shorter hospital stay puts patients at less risk of developing HAI as compared to those who stayed for longer period. This finding was supported by study done in Ethiopia and India [19,42].

Among the 81 patients who developed HAIs 66.7% were inappropriately treated according to IDSA guideline. This was higher than other similar studies [12,46]. This may be due to their study was done on specific illness which was on patients with BSI unlike our study that was done in all HAIs.

The highest inappropriate management of HAI in our study was seen in the department of pediatrics. This was in contrary with study done in Jerusalem [47]. This could be due to the unavailability of more effective first line medications with better safety monitoring procedures in the study setting.

The potential limitation of our study was variables related to health professionals, antiseptics used for patient preparation, methods used for equipment sterilization and type of anaesthesia used were not included due to resource shortage. We were unable investigate the relationship between the adequacy or inadequacy of treatments and clinical outcomes. Comparisons with other studies were difficult due to difference in hospital environment and settings.

In conclusion it was observed that the prevalence of HAI was high in this study. Pneumonia and SSI were the most common type of HAIs followed by UTI. With the limitation in the development of new generations of antibiotics, restrictive and appropriate use of antibiotics is needed to ensure the availability of an effective treatment for hospitalized patients. The higher prevalence of this hospital acquired infection is due to lack of guidelines, lack of standardized infection prevention and antimicrobial stewardship processes in the study area. Antibiotic resistance is a global concern; we recommend that appropriate management of those HAIs with locally conformable guidelines could help to correct such problems and to improve the practice of doing culture and susceptibility test before administration of any antibiotics. Finally the hospital administration should give emphasis for infection prevention practice and establish a member to implement antimicrobial stewardship program.

## Supporting information

S1 File Susceptibility pattern of bacterial pathogens isolated from hospital-acquired infections. (DOCX)

S2 File Key informant interview. (DOCX)

S3 File Data collection tool. (DOCX)

## Abbreviations

ANC: Antenatal Care
AOR: Adjusted Odds Ratio
ASA: American Society of Anesthesiology
BSI: Blood Stream Infection
C&S: Culture and Sensitivity
C/S: Cesarean Section
CAUTIs: Catheter-Associated Urinary Tract Infections
CDC: Center of Disease Control and Prevention
CHF: Congestive Heart Disease
CI: Confidence Interval
CKD: Chronic Kidney Disease
CLABSIs: Central Line Associated Bloodstream Infections
CoNS: Coagulase Negative Staphylococcus
CVC: Central Venous Catheter
DM: Diabetes Mellitus
HAI: Hospital-Acquired Infection
HIV: Human Immune Virus
HTN: Hypertension
ICU: Intensive Care Unit
LP: Lumbar Puncture Procedure
MRSA: Methicilin-Resistant Staphylococcus Aureus
NGT: Nasogastric Tube
NI: Nosocomial Infection
OR: Odds Ratio
P.aeruginos: Pseudomonas aeruginosa
PPI: Proton Pump Inhibitors
PVC: Peripheral Venous Catheter
RTI: Respiratory Tract Infection
S.aureus: Staphylococcus Aureus
SSIs: Surgical Site Infections
SSTIs: Skin and Soft Tissue Infections
TASH: Tikur Anbessa Specialized Hospital
USA: United States of America
UTI: Urinary Tract Infection
VAP: Ventilator-Associated Pneumonia
WHO: World Health Organization
