# Supplementary material for: Management of hospital-acquired infections among patients hospitalized at Zewditu memorial hospital, Addis Ababa, Ethiopia: A prospective cross-sectional study
Source: PLoS One. 2020 Apr 24;15(4):e0231949. doi: 10.1371/journal.pone.0231949 (PMC7182178; doi:10.1371/journal.pone.0231949)
Supplement: S1 File — (DOCX) [file pone.0231949.s001.docx]

**S1 file: Susceptibility pattern of Bacterial pathogens isolated from hospital-acquired**

|  | **Bacterial isolate (n=7)** | | | | | | | | |
| --- | --- | --- | --- | --- | --- | --- | --- | --- | --- |
|  | ***E.Coli* n=4(%)** | | | ***S.aureus* n=2(%)** | | | ***Acinetobacter* n=1(%)** | | |
| **Susceptibility**  **Pattern** | **S** | **R** | **Not done** | **S** | **R** | **Not done** | **S** | **R** | **Not done** |
| **Ampicillin** | - | 4(100) |  | - | - | 4(100) | - | 1(100) |  |
| **Amoxacillin** | - | - | - | - | - | 4(100) | - | - |  |
| **Cefroxime** |  | 4(100) |  | - | - | 4(100) | - | - |  |
| **Ceftriaxone** | 2 (50) | - | 2 (50) | - | - | 4(100) | - | - |  |
| **Ciprofloxacin** | 1(25) | 2 (50) | 1(25) | 2(100) | - | - | 1(100) | - | - |
| **Clindamycin** | - | - | 4(100) | 1(50) | 1(50) | - | - | - | 1(100) |
| **Erythromycin** | - | - | 4(100) | 1(50) | 1(50) | - | - | - | 1(100) |
| **Gentamycin** | 1(25) | 3(75) | 1(25) | 1(50) | 1(50) | - | 1(100) | - | - |
| **Nitrofurantoin** | - | 1(25) | 3(75) | - | - | 2(100) | - | - | 1(100) |
| **Sulfometoxazo** | 1(25) | - | 3(75) | - | - | 2(100) | - | - | 1(100) |
| **Agumentin** | - | 4(100) | - |  |  | 2(100) | - | 1(100) | - |
| **Ceftazidime** | - | 3(75) | 1(25) | 1(50) | - | 1(50) | 1(100) | - | - |
| **Chloramphenic** | 1(25) | 1(25) | 2(50) | 1(50) | 1(50) | - | 1(100) | - | - |
| **Doxycyclin** | - | 3(75) | 1(25) | 1(50) | 1(50) | - | - | - | 1(100) |
| **Oxacillin** | 4(100) | - | - | 1(50) | 1(50) | - | - | - | 1(100) |
| **Meropenem** | 3(75) | - | 1(25) | - | - | 2(100) | 1(100) | - | - |
| **cefepime** | 1(25) |  | 3(75) | - | - | 2(100) | 1(100) | - | - |
| **Erythromycin** | - | - | 4(100) | 1(50) | 1(50) | - | - | - | 1(100) |

R=Resistant, S=Suceptible
