# Supplementary material for: Management of hospital-acquired infections among patients hospitalized at Zewditu memorial hospital, Addis Ababa, Ethiopia: A prospective cross-sectional study
Source: PLoS One. 2020 Apr 24;15(4):e0231949. doi: 10.1371/journal.pone.0231949 (PMC7182178; doi:10.1371/journal.pone.0231949)
Supplement: S2 File — (DOCX) [file pone.0231949.s002.docx]

**S2 file: Key informant interview**

“*I did not send culture and sensitivity test for patients because most of patients will be discharged while waiting for result so I personally prefer to treat empirically also there is shortage of culture material we can’t request when we need it* ” (One of the residents).

“*For patients especially admitted during night time and in the weekends I send sample after treating the patient empirically because the laboratory is closed during night time and in the weekends*”(Three of the resident). “*Sometimes I seek for sending samples after initiating patients on antibiotics when patients didn’t improve”* (five of the residents).

“*The samples received will go through appropriate procedure based on guidelines and with quality control with 98% sensitivity but am doubting the technique of sample collection sometimes skin contaminants are found in blood samples. Regarding the time for culture and sensitivity result on average it took 24-72 hours but sometimes 5-7 days because maximum day for possible fungal or bacterial growth is considered*” (four of the laboratory personnel), (Table 6).

*“We treat empirically and there is irrational antibiotics use that predispose patients for farther HAI development*” (all of the respondents).

“*There is knowledge gap on treating patients I give medication to may patient, if there is no improvement will consult senior physician and sometimes found it that it was not the right choice from the beginning. I tried to check new updates but did not do it regularly due to time constraints*” (five of the residents).

“*Patients sometimes took antibiotics for a long period of time because medications will be added one on top of the other when there is no improvement and we forget to discontinue medications on time*” (two of the residents).

“*I treat patients empirically with lack of supportive laboratory evidences which overestimate the infection prevalence”* (One of the year 3 Resident)

“*Patient flow and inadequate beds especially gyn/obs ward patients are sleeping on corridors predisposing them for infections. Surprisingly patient readmitted after C/s for the diagnosis of SSI she is being treated on the corridor due to lack of bed”* (One of the year 2 Residents).

*“We are doing procedures in dirty environment with full of flies in the operation room even we are forced to give preoperative prophylaxis in case of clean surgery due to fear of infection development”* (One of year 4 resident).

**Addis Ababa University**

**School of graduate studies**

**Department of pharmacology and clinical pharmacy**

Study on the assessment of the management of hospital-acquired infectionsin selected wards of Zewditu memorial hospital.

**Informed consent**

Hello my name is _________________ I am working as a data collector for Miss segen G/meskel from Addis Ababa University, College of Health Sciences, School of Pharmacy, Department of Pharmacology and Clinical pharmacy, Post graduate program. The purpose of this study is to assess the prevalence management of hospital-acquired infectionsin selected wards of Zewditu memorial hospital. Those infections will expose patients to prolonged hospitalization, increased morbidity and mortality. This study may provide information for better control of infections so might benefit you and the society in general through implementing standard procedure to minimize infections. Your participation in this research is entirely voluntary. It is your choice whether to participate or not, all the services you receive at this clinic will continue and nothing will change. And your information will be anonymous. I am kindly inviting you to be part of the research. The research will continue till your discharge period. Also you have a full right to withdraw at any time of the data collection period. If you have any concern you can contact any time through my address.

- Yes
- No Skip to the next person

Sign ______________________ Date____________________

Address of PI

Segen G/meskel

Email: [segengm@gmail.com](mailto:segengm@gmail.com)

Phone: 251-914031220
