# Supplementary material for: Management of hospital-acquired infections among patients hospitalized at Zewditu memorial hospital, Addis Ababa, Ethiopia: A prospective cross-sectional study
Source: PLoS One. 2020 Apr 24;15(4):e0231949. doi: 10.1371/journal.pone.0231949 (PMC7182178; doi:10.1371/journal.pone.0231949)
Supplement: S3 File — (DOCX) [file pone.0231949.s003.docx]

**Data collection tool**

Table 1 **Socio-demographic and clinical characteristics of the study participants**

Patient card NO______Sex: 1. Male 2. Female Age (years)_______ Departme___________ Ward _____________ Admission date__________ Discharge date_________

Reason for admission __________ Previous medical admission 1.Yes 2. No

If yes to the above question, how long was the patient admitted? _____________

Outcome: Discharge__________ Death_________________

Table 2. Risk factors for hospital-acquired infections. Instruction put × in the provided space

| **No** | **Variables** | | **Yes** | **No** | **Duration** |
| --- | --- | --- | --- | --- | --- |
| **1** | Peripheral intravenous line inserted | |  |  |  |
| **2** | Urinary catheter inserted | |  |  |  |
| **3** | Nasogastric Tube inserted | |  |  |  |
| **4** | Use of proton pump inhibitors | |  |  |  |
| **5** | Use of Mechanical ventilator | |  |  |  |
| **7** | Lumbar puncture | |  |  |  |
| **8** | Empiric antibiotic administration other than prophylaxis (specify) | |  |  |  |
| **9** | Chronic illness(Asthma,HTN,DM,IHD) specify | |  |  |  |
| **10** | Use of steroids | |  |  |  |
| **11** | Preoperative prophylaxis(specify) | |  |  |  |
| **12** | If yes, for how long does it continued postoperative? | |  |  |  |
| **13** | Surgery | Elective surgery |  |  |  |
|  |  | Emergency surgery(Day, Night) |  |  |  |
| **14** | Preoperative hospital stay | |  | | |
| **15** | Type of procedure (specify) | |  | | |
| **16** | Duration of procedure | |  | | |
| **17** | Others | |  | | |

**Table 3. Type of infections due to hospital-acquired infections**

| No | Variables | Yes | No |
| --- | --- | --- | --- |
| 1 | Pneumonia |  |  |
| 2 | Urinary tract infection |  |  |
| 3 | Surgical site infections |  |  |
| 4 | Diarrhea |  |  |
| 5 | Bone and joint infection (specify) |  | |
| 6 | Blood stream infection(Sepsis, Meningitis) |  | |
| 7 | Others specify_______________ |  | |

Table 4. Common hospital organisms and their antibiotic susceptibility pattern

| Culture and sensitivity | Drug and susceptibility test (R=Resistant, I=Intermidate, S=Sensitive) | | | | | | | | | | | | | | | | | | | | | | |
| --- | --- | --- | --- | --- | --- | --- | --- | --- | --- | --- | --- | --- | --- | --- | --- | --- | --- | --- | --- | --- | --- | --- | --- |
|  | Ampicillin | Amoxacillin | Cefroxime | Ceftriaxone | Ciprofloxacin | Clindamycin | Erythromycin | Gentamycin | Nitrofurantoin | Sulfometoxazol | Vancomycin | Agumentin | Nalidixio Acid | Ceftazidime | Chloramphenicol | Norfloxacin | Tetracyclin | Doxycyclin | Oxacillin | Kanamycin | Rifampicin |  |  |
|  |  |  |  |  |  |  |  |  |  |  |  |  |  |  |  |  |  |  |  |  |  |  |  |
|  |  |  |  |  |  |  |  |  |  |  |  |  |  |  |  |  |  |  |  |  |  |  |  |
|  |  |  |  |  |  |  |  |  |  |  |  |  |  |  |  |  |  |  |  |  |  |  |  |
|  |  |  |  |  |  |  |  |  |  |  |  |  |  |  |  |  |  |  |  |  |  |  |  |
|  |  |  |  |  |  |  |  |  |  |  |  |  |  |  |  |  |  |  |  |  |  |  |  |

Table 5. Pattern of antibiotic usage for treating hospital-acquired infections

| DX |  | |
| --- | --- | --- |
| Medication PX(full regimen) | 1.  2.  3.  4. | |
| Change of Medication (Full regimen) | After culture | Yes (Specify) |
|  |  | No |
|  | Other reasons(specify) |  |

Key informant interview on management of hospital-acquired infections for Physicians.

First of all, thank you for agreeing to participate on this interview. My name is Segen G/meskel. I will be talking with you today regarding the management of hospital-acquired infections. The main purpose of this interview will be to learn more about the professionals’ existing experience and practice in the wards of Zewditu memorial hospital. The confidentiality regarding the following interview will be strictly maintained. At any time during our interview, please feel free to let me know if you have any questions or we can stop the interview at any time for any reason. The interview will take about 30minutes.

Back ground information

1. Can you tell me your current educational level?
2. How do you manage hospital-acquired infections? Do you follow a specific guideline? If yes, please specify?________________________________________________________________________________________________________________________________________________________________________________________________________________
3. Will you prefer using standard treatment guideline of Ethiopia as a reference? If no why not?________________________________________________________________________________________________________________________________________________________________________________________________________________
4. How did you choose medications to your patients?_______________________________________________________________________________________________________________________________________________________________________________________________________________
5. What are the possible reasons for inadequacy of treatment?___________________________________________________________________________________________________________________________________________________________________________________________________________
6. Why did not you send culture and sensitivity to all patients?_______________________________________________________________________________________________________________________________________________________________________________________________________________
7. What is the reason most of culture results did not show any growth? ____________________________________________________________________________________________________________________________________________________________________________________________________________________
8. Would you depend on results of culture & sensitivity testing for prescription of antimicrobials?___________________________________________________________________________________________________________________________________________________________________________________________________________
9. Why are you giving prophylaxis for most of the procedures?______________________________________________________________________________________________________________________________________________________________________________________________________________
10. Any other points you wish to communicate regarding hospital-acquired infections and its management?_________________________________________________________________________________________________________________________________________________________________________________________________________

Key informant interview for laboratory technicians

1. What can you say about the quality of microbiology laboratory? ______________________________________________________________________________________________________________________________________
2. Why is most culture & sensitivity tests end up with negative result? __________________________________________________________________________________________________________________________________________
3. Who will take the bacteriology test result after it is done? __________________________________________________________________________________________________________________________________________
4. On average, how long does it take for C&S result to come back (in day)? ___________________________________________________________________________________________________________________________________________

Thank you very much for you time and willingness to answer these question.
